# Quantifying central canal stenosis prediction uncertainty in SpineNet with conformal prediction

**DOI:** 10.1038/s41598-026-35343-6

**Published:** 2026-01-10

**Authors:** Andrea Cina, Maria Monzon, Fabio Galbusera, Catherine R. Jutzeler

**Affiliations:** 1 Department of Health Sciences and Technology (D-HEST), ETH Zurich, Universitätstrasse 2, Zürich, 8092 Switzerland; 2 Schulthess Clinic, Department of Teaching, Research and Development, Zürich, Switzerland; 3 Swiss Institute of Bioinformatics (SIB), Lausanne, Switzerland

**Keywords:** Conformal prediction, Uncertainty, Stenosis, SpineNet, Deep learning, Health care, Medical research, Mathematics and computing

## Abstract

This study applies conformal prediction (CP) to SpineNet to quantify prediction uncertainty in the classification of central canal stenosis (CCS) into four grades: normal, mild, moderate, and severe. CP provides prediction sets rather than singleton predictions with a guaranteed probability of containing the true class, offering a transparent way to assess model reliability. This makes CP particularly useful in the medical field, where reliable predictions are critical for decision-making. We analysed 1689 vertebral levels (L1/L2 to L5/S1) from 340 patients who underwent T2-weighted MRI examinations and evaluated four CP methods across multiple significance level. (α). Bootstrap resampling was used to assess robustness across calibration/test splits. Among the evaluated CP methods, the class-conditional CP method consistently achieved the desired coverage while producing the smallest prediction set siss. Top-k produced larger, uninformative prediction sets (~ 4), and Least Ambiguous Set-Valued Classifiers (LAC) and Adaptive Prediction Sets (APS) showed reduced performance, particularly in moderate and severe cases. A class- and level-specific analysis with class-conditional CP (α = 0.15) revealed smaller prediction sets for normal and mild grades (~ 1.5) and larger sets (~ 2.7–3) for less frequent moderate and severe grades, reflecting higher uncertainty in these categories. Overall class-conditional CP emerged as the most reliable and clinically informative approach for estimating uncertainty in CCS grading.

**Supplementary Information:**

The online version contains supplementary material available at 10.1038/s41598-026-35343-6.

## Introduction

In recent years, artificial intelligence (AI) has been transforming healthcare, driven by the abundance of available data^1^. The rapid development of models capable of analysing images has significantly impacted medical image analysis^2^. Convolutional neural networks (CNNs), a specific type of neural network architecture, have been widely used for tasks such as image classification^3,4^, object detection^5,6^, and segmentation^7^. The field of spinal imaging is no exception to this trend. Several research groups have implemented deep learning (DL) models to classify different spinal degenerative conditions and fractures^8,9^, segment spine segments^10^, and automatically label vertebral bodies to compute a set of spinal parameters^11,12^. One of the most recognised models in the spine field is SpineNet^13^. SpineNet was trained on T2-weighted MRIs from patients recruited as part of the GENODISC consortium project^14^ at multiple centres in the United Kingdom, Hungary, Italy, and Slovenia using a range of different MRI scans and acquisition protocols. SpineNet automatically detects and labels vertebral bodies in sagittal MRI and performs radiological grading of each intervertebral disc level in T2-weighted lumbar scans from L2/L1 to S1/L5. The grading includes among others the 5-class Pfirrmann grading^15^ and the 4-class central canal stenosis (CCS) according to the Lee grading system^16^.

SpineNet was externally validated by two independent studies: a group from Finland evaluated SpineNet performance on 1331 patients, finding robust results despite some challenges in generalising to external datasets^17^. Similarly, a study in Switzerland assessed SpineNet on two datasets comprising 882 patients, reporting comparable findings^18^. Despite these advancements, ensuring consistent accuracy across diverse clinical settings remains crucial for integrating such models into clinical workflows when applied to data with distributions different from those seen during training^19^. As a result, estimating prediction uncertainty is essential for detecting unreliable outputs. However, DL models typically provide only single-point predictions, without an associated measure of prediction reliability or uncertainty.

In practice, they assign a single class label to each case (e.g., “class A”), whereas considering multiple possible classes with a known confidence level can be more valuable for clinical decision-making. Conformal Prediction (CP) addresses this limitation by offering a rigorous statistical framework for uncertainty quantification in prediction tasks^20,21^. Specifically, CP enhances DL models by providing a set of possible classes while guaranteeing that the correct class will be included in the prediction set with a user-specified probability (coverage)^20,21^. The coverage is given by the confidence level 1 - α, where α is the user-defined error tolerance. A smaller α increases coverage but typically results in larger prediction sets, meaning more classes are included in the output. The key advantage of CP lies in its distribution-free validity guarantees and its ability to integrate seamlessly with existing models.

Until recently, CP has been largely overlooked in the medical field, with only a few studies incorporating this framework into their models. Lu et al.^22^ applied various CP methods to a dermatology photography dataset for skin lesion classification. Another study utilised CP to enhance an in-house model for classifying prostate biopsies as benign or malignant^23^. In spinal imaging, to the best of our knowledge, only one study has applied CP to MRI classification of degeneration^24^. More recently, some works have introduced Monte Carlo CP (MCCP), an extension of standard CP methods designed to account for the ambiguity and uncertainty around the ground truth labels themselves^25,26^. The idea is that, usually, when multiple raters are involved, the ground truth label is assigned using a majority voting approach without considering the distribution of the votes. With MCCP, this votes distribution is modelled during the calibration process to inform the calibrated threshold computation.

The present study evaluates four CP methods applied to the pre-trained SpineNet model to quantify uncertainty in CCS classification using an external dataset of T2-weighted sagittal lumbar MRIs. The analysis focuses on CCS classification across four severity grades (normal, mild, moderate, and severe)^16^. The four methods were selected based on their ease of implementation and simplicity as the present study should be accessible also to a non-technical readership. Moreover, the four methods are widely used in the CP community and they have been implemented in the most common CP libraries such as MAPIE^27^. The primary goal is to examine how different alpha levels impact prediction set sizes, helping clinicians select appropriate confidence thresholds for various clinical scenarios. Additionally, multiple dataset resamplings are used to assess the robustness of the approach and establish confidence intervals for both coverage and prediction set sizes. Finally, an in-depth analysis explores how coverage and prediction set sizes vary across CCS classes at different vertebral levels.

## Results

The CP framework was implemented for four-class CCS predictions using the SpineNet model on a dataset of patients with lumbar stenosis from our hospital in Switzerland. The dataset contained 340 patients with a mean age of 72 years (SD = 8.8), of which 42% were males. SpineNet automatically graded up to five levels per patient (L2/L1 to S1/L5). After excluding undetected levels (11), the final analysis comprised 1689 vertebral level instances. Across the four CCS categories, mild stenosis was the most common, accounting for 46% of cases, followed by severe (20%), moderate (18%), and normal (16%). To integrate CP, we extracted the softmax outputs from SpineNet’s final layer for each image, providing probability estimates for the four stenosis grades. These probabilities were then used within the CP framework.

**Fig. 1:**
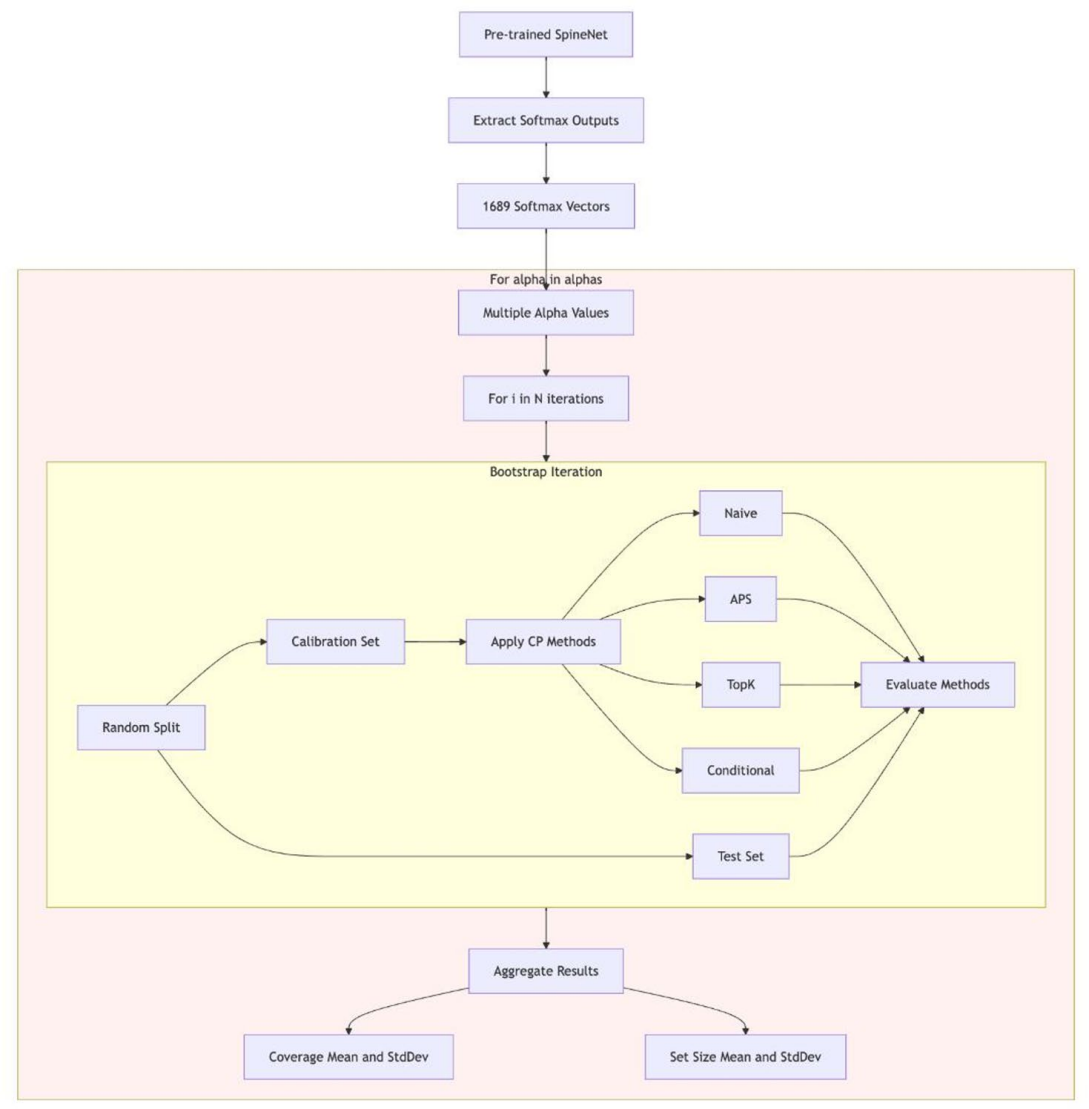
Workflow of the proposed approach. SpineNet model extracts softmax outputs from 1689 cases. Multiple error rate levels α were evaluated. For each α value, N bootstrap iterations are performed, where each iteration randomly splits the data into calibration and test sets. Four CP methods (LAC, APS, Top-k, and class-conditional) are applied to the calibration set and evaluated on the test set. The framework aggregates results across all bootstrap iterations to compute statistics of both prediction set sizes and empirical coverage for each significance level.

**Fig. 2:**
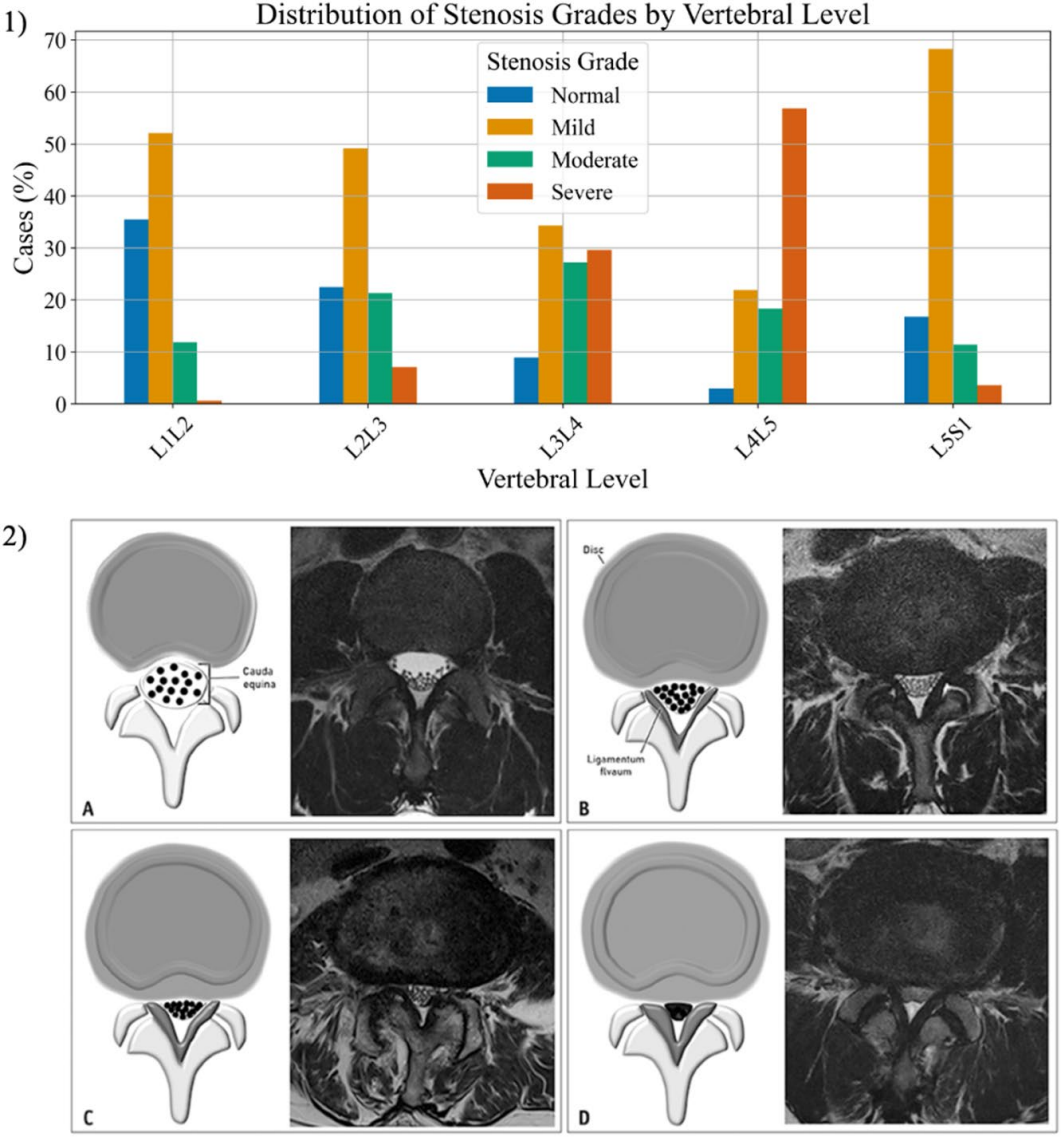
Distribution of stenosis gradings across vertebral levels and appearance of Central Canal Stenosis grades. (1) Mild and moderate cases are well represented across all the vertebral levels. Severe cases are mostly in L3/L4 and L4/L5 levels. Normal stenosis is mostly in the upper lumbar levels. (2) In the second panel, how the stenosis grades appear: (**a**) Normal grade, (**b**) mild grade, (**c**) moderate grade, and (**d**) severe grade. The severity of stenosis increases with the compression of the Cerebrospinal fluid.

Since SpineNet was trained on a completely separate dataset, a random 50/50 split into calibration and test sets keeping the proportion of the classes across the two sets was used. Additional results with a 70/30 ratio to investigate the influence of using a larger calibration set are reported in the supplementary material (see Supplementary Figs. S1, S2, and S3 online). The 50/50 split guarantees that the calibration set is sufficiently large to compute a robust CP threshold while still preserving enough data to effectively evaluate the approach. The split was repeated multiple times (1000 bootstrapping iterations) to assess the robustness and stability of the approach across different splits of the dataset. Four different CP techniques were applied to the calibration set: Least Ambiguous Set-Valued Classifiers (LAC)^28^, Adaptive Prediction Sets (APS)^29^, top-k CP^29,30^, and class-conditional CP with class-specific thresholds^31^. After bootstrapping, the results were aggregated to estimate confidence intervals of the empirical coverage and the prediction set sizes (Fig. 1). Empirical coverage and prediction set size are the two primary metrics used to evaluate CP. To assess the impact of different error tolerance levels (α = 0.05, 0.1, 0.15, and 0.2), bootstrapping and result aggregation were performed across multiple iterations. The evaluation of CP methods was conducted at three levels: (1) overall performance metrics across all classes, (2) class-specific analysis for each CCS grade, and (3) a combined class- and vertebral level-specific analysis to examine variations in coverage and prediction set size across different vertebral levels. The latter was particularly relevant, as stenosis grades are distributed unevenly across levels (Fig. 2 top).

### Overall evaluation

Regarding overall metrics, empirical coverage closely matched the expected (1 - α) across all alpha levels. While top-k CP consistently showed the highest coverage, this came at the cost of large prediction set sizes.

**Fig. 3:**
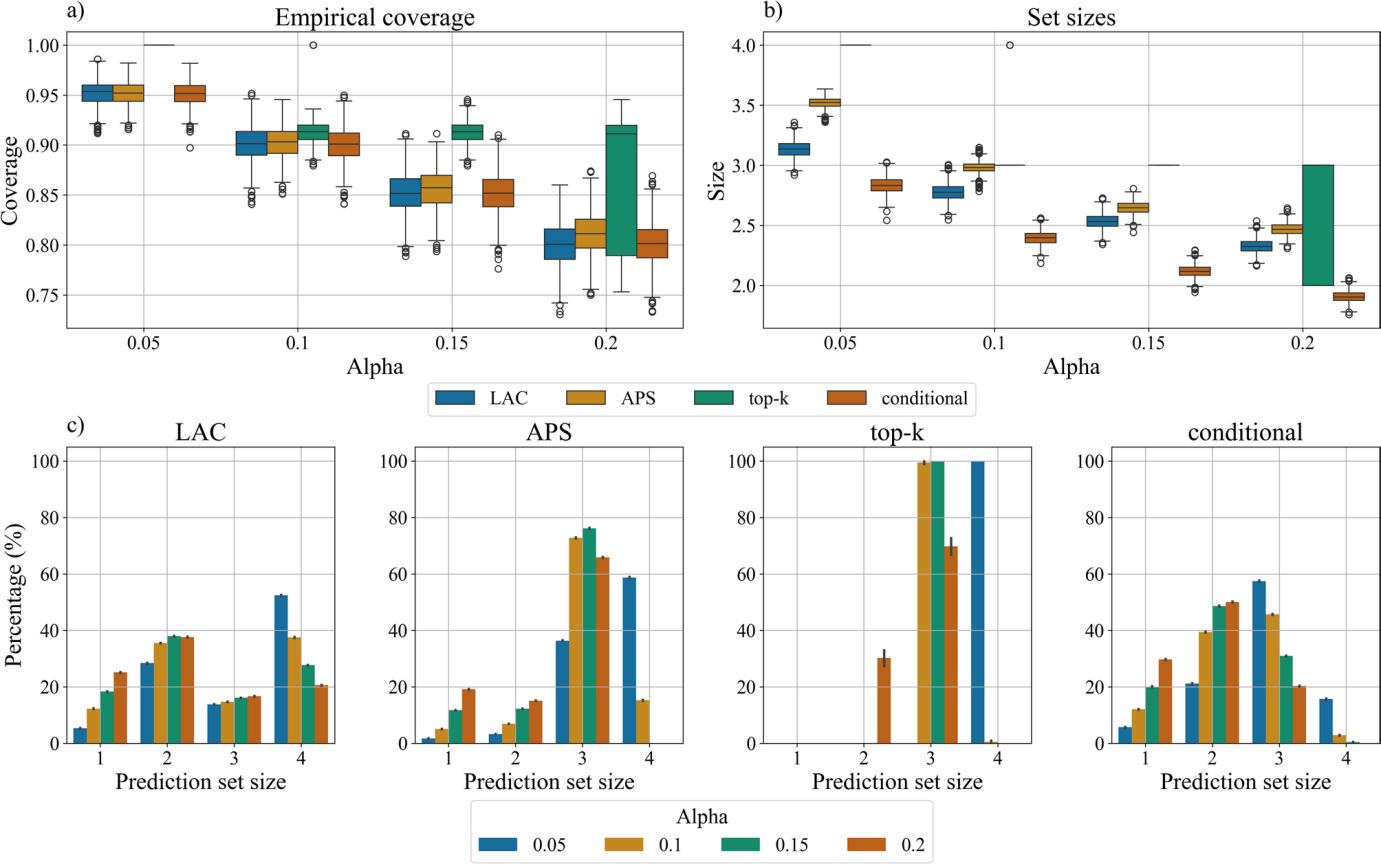
Overall metrics. (**a**) Empirical coverage achieved by LAC, APS (Adaptive Prediction Sets), top-k, and class-conditional methods for α values ranging from 0.05 to 0.2. The box plots show the distribution of coverage values, with higher values indicating better performance. (b) Average prediction set sizes for each method across different α values. (**c**) Distribution of prediction set sizes across different conformal prediction methods at varying significance levels (α). Each subplot represents a different method: LAC (Locally Adaptive Conformal), APS (Adaptive Prediction Sets), top-k, and class-conditional conformal prediction. The bars show the percentage of samples receiving each prediction set size (1–4), with error bars indicating uncertainty. Higher α values generally lead to smaller prediction sets, though the pattern varies significantly across methods.

Notably, at α = 0.05, top-k CP reached 100% coverage but included all four classes in its prediction sets, rendering it non-discriminative and clinically impractical. At other significance levels except α = 0.2, top-k maintained higher than expected coverage but continued to produce large prediction sets. The remaining methods demonstrated stable performance, with coverage consistently matching the expected coverage and showing minimal variability around the median (Fig. 3a). However, prediction set sizes were more variable across the different methods (Fig. 3b).

Excluding top-k that was already described, APS had the highest sizes across all α levels ranging from 2.47 (95% CI: 2.43–2.52) for α = 0.2 to 3.51 (95% CI: 3.37–3.58) for α = 0.05.

The class-conditional CP method was the most balanced approach, achieving expected coverage levels while maintaining the smallest prediction set sizes (Fig. 3). Specifically, it achieved empirical coverages of 0.95 (95% CI: 0.93–0.97), 0.90 (95% CI: 0.88–0.93), 0.85 (95% CI: 0.83–0.89), and 0.80 (95% CI: 0.78–0.84) for α values of 0.05, 0.1, 0.15, and 0.2 respectively. The corresponding prediction set sizes were 2.87 (95% CI: 2.71–2.98), 2.42 (95% CI: 2.32–2.57), 2.13 (95% CI: 2.02–2.26), and 1.93 (95% CI: 1.85–2.06).

The percentage of samples for each prediction set size was also investigated (Fig. 3c). This analysis gives additional insight into each algorithm’s uncertainty quantification approach. LAC shows a more balanced percentage across sizes, with most predictions containing 2–4 classes, while APS and top-k have sets of size three (more than 70–80% of the predictions). Top-k has also a lot of size four prediction sets without any single class predictions. The class-conditional CP method demonstrates the most uniform distribution with very few prediction sets of size four indicating more confidence in the predictions.

In particular, for α = 0.15 and α = 0.2, most of the predictions (> 60%) had prediction set size of 1 or 2 (Fig. 3c last plot on the right green and red bars).

### Stenosis grades evaluation

**Fig. 4:**
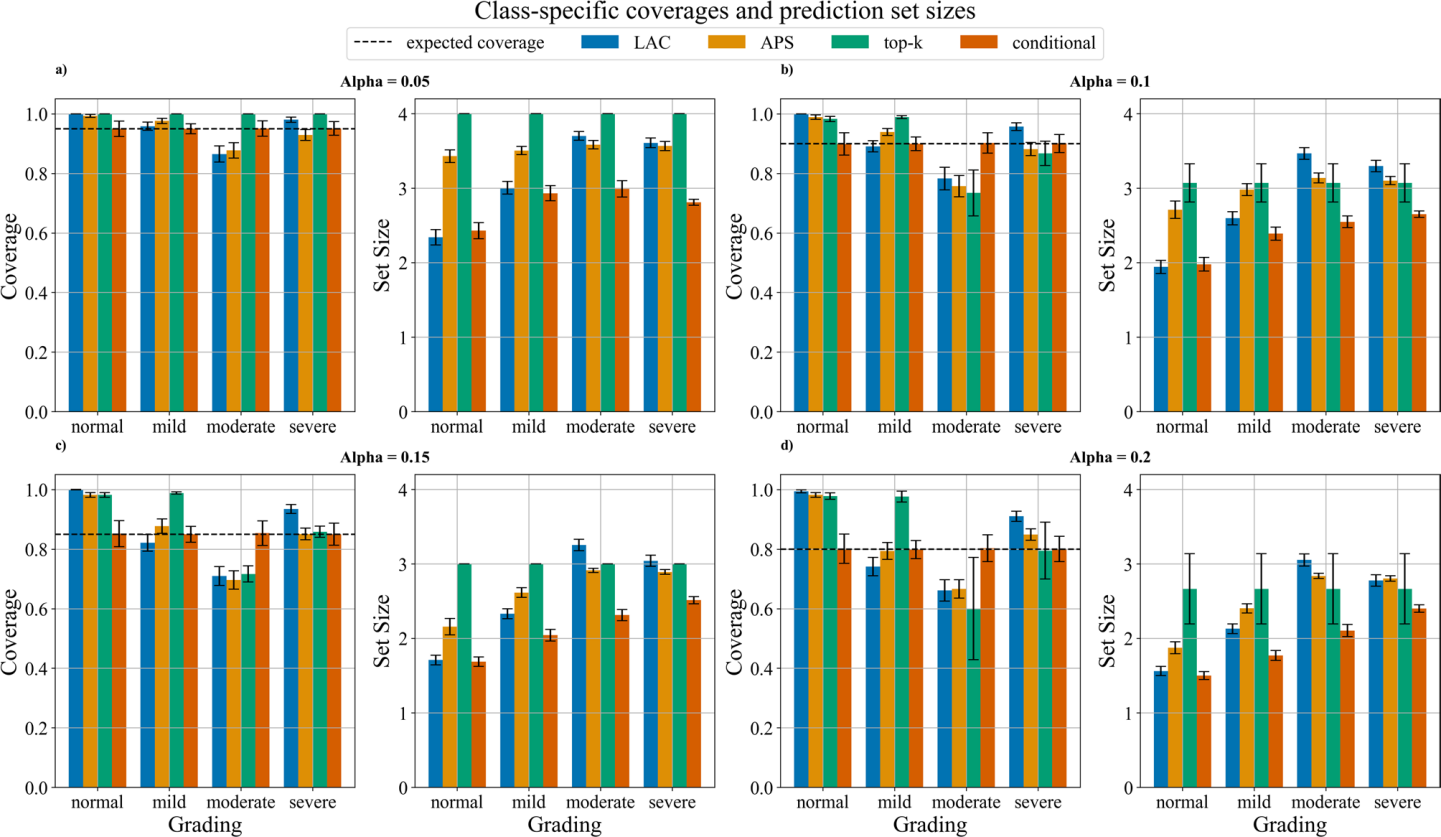
Class-specific coverages and prediction set sizes. Each subplot pair shows the class-conditional coverage (left) and corresponding prediction set sizes (right) for different stenosis grades (normal, mild, moderate, and severe) at: (**a**) α = 0.05, (**b**) α = 0.1, (**c**) α = 0.15, and (**d**) α = 0.2. Four methods are compared: LAC (blue), APS (orange), top-k (green), and class-conditional (red). The dashed horizontal line in the coverage plots represents the expected coverage level (1-α). Error bars indicate 95% confidence intervals. The prediction set sizes show the average number of predictions needed to achieve the corresponding coverage levels for each method and stenosis grade.

For class-specific evaluation of the CCS at α = 0.05, top-k achieved perfect coverage (100%) across all classes. However, this came at the cost of consistently requiring prediction sets of size four. Looking at the other methods for α = 0.05, the class-conditional CP method was the best performing showing high coverage (0.95) with narrow confidence intervals across all stenosis gradings and the smallest prediction set sizes ranging from 2.49 (95% CI: 2.34–2.61) for normal to 3.00 (95% CI: 2.80–3.17) for moderate stenosis (Fig. 4a). LAC and APS methods did not achieve the expected coverage level for moderate grading with empirical coverage of 0.87 (95% CI: 0.83–0.90) and 0.86 (95% CI: 0.81–0.90) respectively. Additionally, APS did not reach the expected coverage for severe cases achieving only 0.92 (95% CI: 0.89–0.95) (Fig. 4a left). For α = 0.1, class-conditional CP maintained its superior performance consistently achieving the expected coverage for all the stenosis gradings with the smallest prediction set sizes (Fig. 4b). In particular, the prediction set sizes ranged from 2.02 (95%CI: 1.92–2.13) for normal to 2.66 (95% CI: 2.60–2.74) for severe stenosis. LAC, APS, and top-k did not achieve the expected coverage for the moderate class with confidence intervals not crossing the 0.9 line (Fig. 4b left) despite the large prediction set sizes (Fig. 4b right). APS and top-k upper confidence intervals are just above the expected coverage for severe cases despite prediction set sizes of around 3.

For α = 0.15 (Fig. 4c), the top-k method demonstrated high coverage (> 95% vs. 0.85 expected) for both normal and mild cases, though with notably larger prediction set sizes (size 3) compared to other methods. For moderate stenosis, top-k’s performance dropped significantly to around 0.71 coverage, well below the expected coverage of 0.85. The class-conditional CP method maintained consistent coverage across all stenosis grades, with prediction set sizes ranging from 1.49 (95% CI: 1.39–1.60) for normal to 2.39 (95% CI: 2.29–2.49) for severe stenosis. Both LAC and APS methods struggled to achieve expected coverage for moderate stenosis, with coverage values around 0.71 (95% CI: 0.66–0.79) and 0.69 (95% CI: 0.64–0.74) respectively. For α = 0.2 (Fig. 4d), similar patterns emerged but with generally lower coverage values as expected due to the higher significance level. The top-k method showed strong performance for normal and mild cases (> 95% coverage) but suffered a drop in coverage to approximately 0.58 (95% CI: 0.36–0.75) for moderate stenosis. The class-conditional CP method maintained the most stable performance across all stenosis grades, achieving the expected coverage of 0.8 consistently. Prediction set sizes for the class-conditional CP method ranged from about 1.53 (95% CI: 1.47–1.61) for normal to 2.44 (95% CI: 2.36–2.63) for severe stenosis. LAC and APS methods continued to underperform with moderate stenosis, showing coverage values of 0.67 (95% CI: 0.62–0.76) and 0.66 (95% CI: 0.61–0.70) respectively, significantly below the expected coverage level.

### Vertebral levels evaluation

The vertebral level-specific analysis was computed using the best-performing CP approach, namely class-conditional CP, with an acceptable alpha level of 0.15 to guarantee a 0.85 coverage. L1/L2 level was mostly affected by Normal and Mild grades with very few severe cases, while L4/L5 shows the highest percentage of Severe stenosis around 55% (Fig. 2 top).

**Fig. 5:**
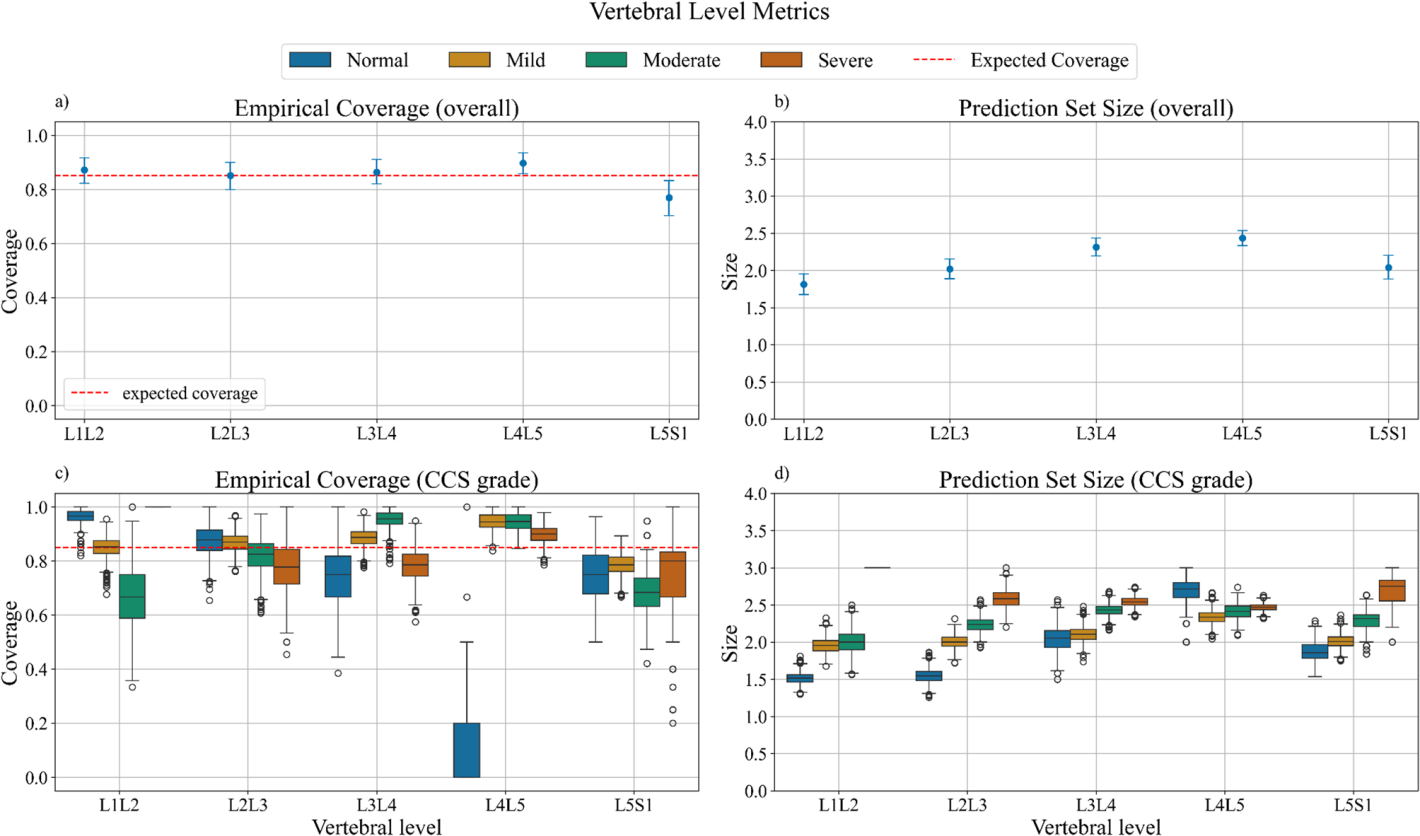
Empirical coverage and set sizes for each vertebral level and for each vertebral level stratified according to the stenosis grade using the class-conditional method. (**a**) Mean and confidence intervals of the empirical coverage across the vertebral levels. The red dashed line is the expected coverage (0.85). (**b**) Mean and confidence intervals of prediction set sizes across all levels. (**c**) Empirical coverage for each level stratified by stenosis grade (represented with different colors). The black dashed line is the expected coverage. (**d**) Prediction sets sizes for each level stratified by stenosis grades.

Moving down the spine from L1/L2 to L4/L5, there is a general trend toward increasing stenosis severity, though L5/S1 shows the opposite behavior with mild stenosis being the most common (around 70%). Mild stenosis is consistently present across all levels, but its proportion varies significantly. The mean overall empirical coverage over 1000 bootstrap iterations was always greater than or equal to the expected (0.85) except for L5/S1 level where it dropped to 0.77. The mean prediction set sizes ranged from 1.82 for L1/L2 level to 2.44 for L4/L5 (Fig. 5a and b).

Empirical coverage and prediction set sizes were further analysed across vertebral levels and stenosis grades (Fig. 5c and d). Coverage for the normal grade consistently exceeded the expected coverage (0.85) across most levels, such as L1/L2 and L2/L3 but had higher variability at L4/L5, where normal cases only represented 5% of the cases.

Mild grade achieved consistent coverage close to 0.85 at L1/L2, L2/L3, and L5/S1 levels where it is the most prevalent (40–50%) but showed higher variability at levels with fewer mild cases (L4/L5).

Moderate stenosis demonstrated greater variability, particularly at L4/L5 and L5/S1, where it constitutes only 20% of the cases, but the median coverage aligned with the expected value at many levels. The severe grade, being the least frequent (≤ 10% at most levels), showed substantial deviations from the expected coverage, particularly at L3/L4 and L4/L5, highlighting the challenge of achieving consistent performance for underrepresented grades.

Regarding prediction set size, the smallest sets were observed for normal and mild grades, indicating higher confidence in predictions. For instance, the median prediction set size for normal and mild was around 1.5 for L1/L2 and L2/L3 levels. On the other hand, moderate and severe had larger prediction set sizes with a maximum of size three for level L1/L2.

However, for L3/L4 and L4/L5 where they were more represented the prediction set sizes were around 2.5. Severe grade cases showed the largest sizes indicating high uncertainty in the prediction.

## Discussion

This study presents a clinical application of the CP framework to investigate uncertainty estimation in CCS grading. Specifically, by taking advantage of the SpineNet model^13^ to compute the softmax outputs of CCS gradings, four different CP methods were applied to build prediction sets for each sample and investigate the uncertainty of the model in CCS grading. Multiple error rate levels α were tested to understand their relationship with empirical coverage and prediction set size.

Our results demonstrate that while all methods generally achieved the expected marginal coverage, their performance varied significantly across different stenosis grades and α levels.

The class-conditional CP method consistently emerged as the most balanced approach, maintaining expected coverage overall and across all stenosis grades while requiring smaller prediction set sizes compared to other techniques (Figs. 3 and 4, and 5). This was particularly evident in the challenging moderate stenosis cases, where other methods frequently struggled to achieve expected coverage. This happens because class-conditional CP learns a separate threshold for each class. Rare, difficult classes may need a higher threshold, but this only affects those few cases, so common classes still get small prediction sets.

The stability of the class-conditional CP method across different α values (from 0.05 to 0.2) highlights its potential for clinical applications where varying levels of confidence might be required for different diagnostic scenarios. This stability paired with high coverage and small prediction set size is also an indicator of higher confidence in the prediction. Although top-k achieved perfect coverage in some cases, particularly at lower α values, this came at the cost of large prediction sets, often including all possible classes and thus providing limited clinical value (Figs. 3 and 4). Both LAC and APS methods showed significant limitations in handling moderate stenosis cases, with coverage values not achieving target levels, especially at higher α values (Fig. 4).

These findings highlight the importance of careful method selection when implementing uncertainty quantification in medical applications. The robust performance of the class-conditional CP method, characterised by consistent coverage and minimal prediction set sizes, suggests its suitability for clinical implementation where both reliability and specificity are crucial for decision-making. Although class-conditional CP performed best in our setting, the other approaches may be suitable in different scenarios, for example, when class distributions are more balanced. Moreover, LAC and APS can provide more stable results if one class is heavily underrepresented, meaning that there is a very small number of samples for a specific class to compute a reliable threshold.

Since the class-conditional CP method was outperforming the others, a vertebral level-specific analysis for each stenosis grade was performed using α = 0.15. The relationship between grade distribution and performance metrics is evident. Levels like L1/L2 and L2/L3, dominated by mild cases (~ 50%), exhibited stable coverage and small prediction set sizes for this grade. Conversely, levels such as L4/L5 and L5/S1, where the distribution of mild and moderate grades was lower (30–40% each), showed greater variability in both coverage and prediction set size. Underrepresented grades, such as severe stenosis, which account for less than 10% of cases at most levels, presented the most significant variability in coverage and larger prediction set sizes, emphasizing the sensitivity of the method to class imbalance.

These results demonstrate how the conformal prediction approach adapts to varying levels of uncertainty across stenosis grades and vertebral levels.

However, the observed variability in underrepresented grades and vertebral levels underscores the challenge of maintaining consistent performance in the presence of class imbalance. Moreover, the better performance of the class-conditional method is likely influenced by the low recall of the base model in the mild and moderate classes, as its class-specific thresholds mitigate the effects of class imbalance. Despite this, the class-conditional approach offers clinicians valuable uncertainty information while preserving practical utility in the diagnostic workflow. Although the choice of α and the CP method depends on the context and acceptable risk of missclassification, some general recommendations can be made. The significance level α determines the accepted error rate, and values between 0.05 and 0.15 typically offer a good balance between coverage and prediction set sizes in diagnostic settings. Lower α gives more conservative, larger sets, while higher α provides narrower sets at the cost of reduced coverage. For methods, class-conditional CP is preferable when class imbalance is present, as in CCS grading, because it maintains a more stable coverage across classes. LAC and APS may be useful when classes are more evenly distributed.

Importantly, this study contributes to the growing application of CP combined with pre-trained models in radiological imaging, offering valuable insights into uncertainty quantification for clinical decision support. Only a few studies have applied CP in the medical field^22,23,32^, and just one has explored its use in spinal imaging^24^.

Compared to previous work in the spine field^24^, our approach demonstrated more reliable empirical coverage for the class-conditional CP method, aligning more closely with expected values and improving the inclusion of the true class in the prediction set. While the highest coverage was achieved, it came at the cost of larger prediction set sizes, particularly for severe cases.

A notable limitation of the present study is that CP was applied to a pre-trained model. SpineNet was directly used on an MRI dataset with a different data distribution from the images on which it was trained. In particular, prediction set size is affected by the performance of the model because a poor-performing model would always produce large prediction sets to achieve the expected coverage set by the user. In particular, the accuracies of SpineNet on our dataset were 0.95, 0.12, 0.13, and 0.48 for normal, mild, moderate, and severe grades respectively. Notably, while the accuracy for mild and moderate grades was low, most misclassifications occurred between adjacent classes (e.g., 85% of mild cases were classified as normal). Our approach mitigates such errors by assigning an image to multiple classes when the model is uncertain about its prediction. Nevertheless, having a larger dataset for training on proprietary data would improve the reliability of the CP approach by producing narrower prediction sets. An alternative strategy could be fine-tuning the model on a subset of the available data to better align its softmax predictions with the specific dataset being analysed. However, the proposed CP-based approach remains a valuable alternative in scenarios where computational resources and technical expertise for training or fine-tuning personalised models are limited. Researchers only need a pre-trained model to generate softmax predictions, which can then be processed using CP to gain insights into challenging cases through coverage and prediction set size analysis.

Another limitation is that the conformalisation did not use the ordinality of the CCS grades. Standard CP methods treat the outcome as nominal, which means the resulting prediction sets may include non-consecutive classes. Ordinal extensions of CP could mitigate this by inducing prediction sets reflecting the ordered structure of stenosis severity. Although these methods exist, they are more difficult to implement and they would likely benefit from an underlying ordinal model. Since SpineNet was originally trained using a standard multiclass classification loss, we used conventional CP methods for simplicity. Implementing ordinal-aware CP might be a promising future direction.

An additional consideration relates to class prevalences. Although detailed class proportions of the dataset used to train SpineNet are not reported^13^, our dataset shows very similar proportions to the one described in the Radiological Society of North America (RSNA) Lumbar Degenerative Imaging Spine Classification (LumbarDISC) dataset^33^. More generally, differences in class proportions across datasets represent a common form of distribution shift in medical imaging. While standard CP does not explicitly model prevalence, this does not invalidate its utility: when the underlying model encounters a shift in class distribution, its predictive performance declines, and CP should appropriately reflect this through larger prediction sets. Beyond class prevalence, other forms of distribution shifts, such as differences in age, gender distribution, and ethnicity might also impact the CP. These differences could also be present between calibration and test sets. However, this assumption was assessed, as conformal prediction requires the calibration and test sets to be exchangeable. Thus, CP can provide a transparent indication of reduced confidence under distribution shifts, which is valuable for clinical decision-making.

In conclusion, this study presents an easy-to-implement approach to estimate uncertainty in predictions agnostic to complex DL models. The simplicity of the proposed method makes it understandable to non-technical users, such as clinicians, by providing a clear and practical application of the CP framework to their field. By raising awareness of these tools, we aim to encourage their wider adoption in real-world clinical settings, where reliable uncertainty estimation can support more informed decision-making.

## Methods

The proposed approach aims to provide a complete workflow that can be used in different scenarios to estimate model prediction uncertainty. The pipeline is very flexible offering a model-agnostic approach that can be integrated with both pre-trained models and custom models trained on proprietary datasets. Through CP techniques, the framework enables researchers to identify uncertain predictions and flag challenging cases, making it valuable for medical settings where reliability is crucial.

### Dataset

MRI scans from two previously approved clinical studies were used: Lumbar Spinal Stenosis Outcome Study (LSOS)^34^, focusing on lumbar spinal stenosis, and the Appropriateness of Surgery study^35^, examining lumbar degenerative spondylolisthesis. The analysis included patients scheduled for either conservative or surgical treatment. Patients were eligible if they were 18 years or older and provided informed consent. Patients with scoliosis deformity exceeding 15°, cauda equina syndrome, acute fractures or infections, isthmic/lytic spondylolisthesis, confirmed peripheral vascular disease, or insufficient language proficiency to complete the study questionnaires were excluded. The use of the data was approved by the ethical committee (IRB: Ethics committee approval, KEK-ZH-2023-01683) and all research was performed in accordance with relevant guidelines.

### SpineNet

SpineNet V2^13^, a convolutional neural network designed to automatically grade multiple radiological conditions from MRI scans, was used for this study. Among these features, SpineNet evaluates CCS, which represents the narrowing of the central canal at the intervertebral disc level. CCS is graded on a four-point scale according to the Lee grading system^16^: grade 0 (normal), grade 1 (mild), grade 2 (moderate), and grade 3 (severe) stenosis (Fig. 2 bottom). SpineNet was applied to the MRI dataset of 1689 vertebral level images to extract the softmax outputs that are needed to apply CP. The softmax output represents the model’s predicted probabilities for each stenosis grade, expressed as a 4-element vector where the first position indicates the probability the model assigns to normal, the second position is the probability the model gives to mild stenosis and so forth for the remaining grades. A dataset with 1689 rows (vertebral levels) and 4 columns (estimate of softmax probabilities) resulted after applying SpineNet.

### Conformal prediction

**Fig. 6:**
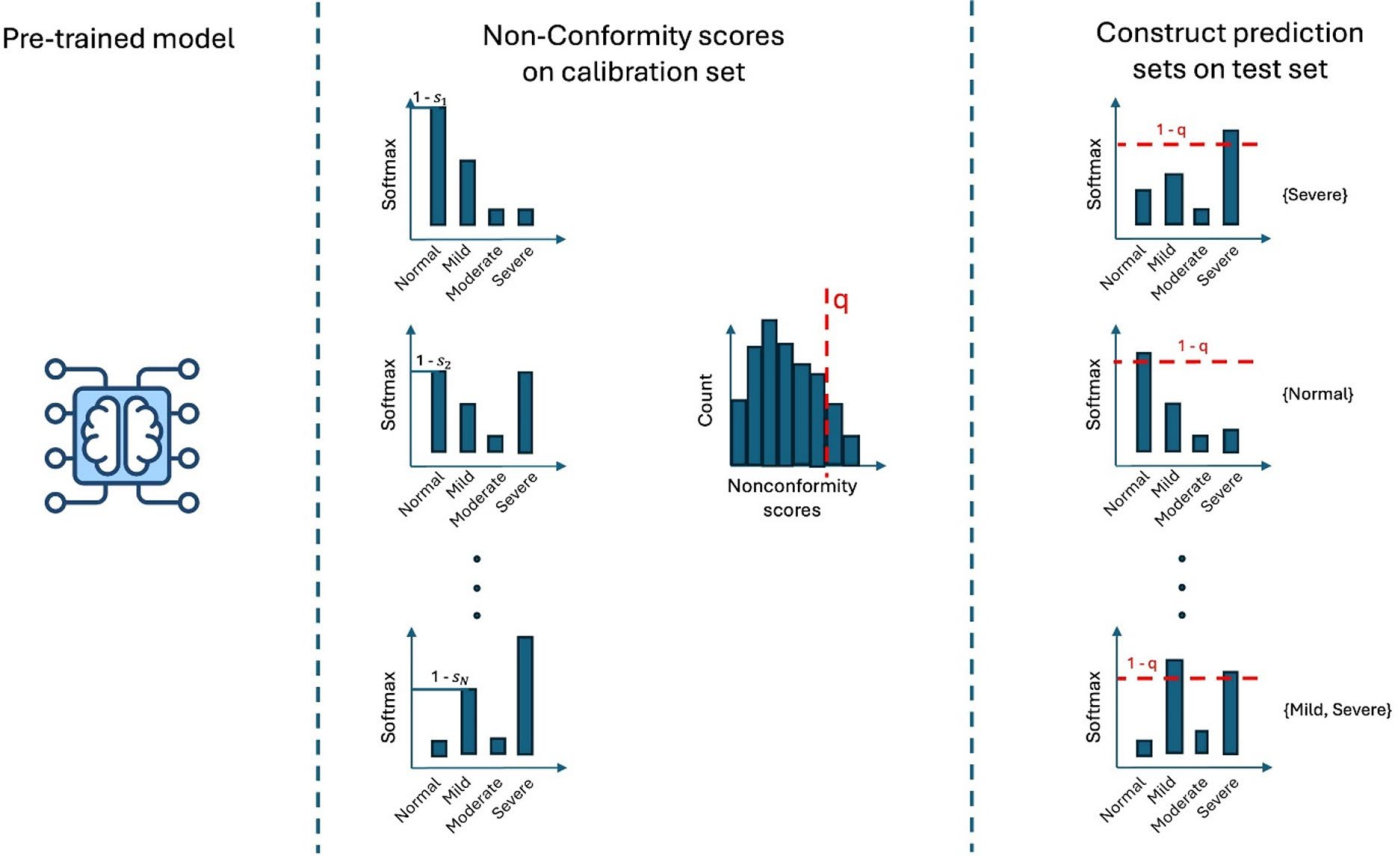
Generic conformal prediction pipeline. First, a pre-trained model is applied to a calibration set, and nonconformity scores (e.g., 1 − softmax probabilities) are computed for each sample (middle panel). From the distribution of these scores, a chosen quantile q (1 - α) becomes the threshold for constructing prediction sets. In the final step, this threshold is applied to new (test) samples, generating sets of potential labels (right panel) that reflect varying levels of uncertainty (e.g., “Normal,” “Normal or Mild,” or “Normal, Mild, or Severe”). Different conformal prediction methods may use slightly different strategies for computing nonconformity scores and determining the quantile, but the overall process remains the same.

CP is a simple way to turn any complex ML or DL model into a framework that provides reliable uncertainty estimates and guarantees on the validity of its predictions^20,21^. The idea is to start with a fitted model, which can be a pre-trained model or one trained from scratch if sufficient training data is available. Before applying CP it is needed to set a desired error rate α such that the true label lies within the prediction set with 1 - α probability (coverage). For example, if α = 0.1, CP guarantees that the true label will lie within the prediction set at least 90% of the time. The coverage is marginal, meaning that, on average, across all samples, the probability is 90%. However, it may be lower for specific subgroups of patients as conditional coverage is not guaranteed. Then a calibration set is required to implement CP. This is a subset of data, separate from the training set, used to compute the non-conformity scores that capture how uncertain the model is about each prediction. In the context of classification, the most common non-conformity measure is 1 — predicted class probability (softmax output of the true class) for the given label. So, if the predicted probability of a new instance belonging to a certain class is high, the non-conformity score will be low, and vice versa. These scores are computed on every instance of the calibration set to create a distribution and then the 1 - α quantile is taken as the threshold to apply to the test set. Thus, once the CP method is applied to the calibration set, the resulting threshold is used to generate prediction sets on the test set, providing uncertainty estimates for each prediction (Fig. 6).

### Pipeline

After extracting the softmax outputs from SpineNet, to ensure robust evaluation, multiple significance levels (α = [0.05, 0.1, 0.15, 0.2]) and 1000 bootstrap iterations for each α value were implemented. In each iteration, the dataset was randomly split into calibration (50%) and test (50%) sets, maintaining class proportions and ensuring that the vertebral levels of the same patients were present only in one of the two sets to avoid data leakage. The calibration set was used to learn appropriate thresholds for creating prediction sets, while the final prediction was performed on the test set. Four conformal prediction methods were evaluated: LAC conformal prediction^28^, APS^29,30^, top-k CP^30^, and class-conditional conformal prediction with class-specific thresholds.

### LAC CP

LAC CP is the simplest method. For each sample in the calibration set, we computed a non-conformity score using the softmax probability assigned to the true class. The threshold to construct the prediction set on the test set is obtained from the 1- α quantile of these non-conformity scores. For a test sample, the model is first applied to compute softmax probabilities of all classes. Then the conformity scores are computed and a test sample is assigned a prediction set containing all classes whose non-conformity scores fall below the calibrated threshold. This provides a straightforward way to estimate uncertainty without additional model assumptions.

### APS CP

APS builds upon the LAC approach by considering the cumulative distribution of the non-conformity scores. In particular, for each sample in the calibration set, classes are ranked in descending order of softmax probability, and a cumulative non-conformity score is computed by sequentially summing the probabilities of the ranked classes until the true class is included. The 1- α quantile of these cumulative scores is used as the calibration threshold similar to LAC. For a test sample, the model’s softmax probabilities are again sorted in decreasing order. The probabilities are summed following the order, and the classes are included in the prediction set until the quantile is reached. APS typically produces more compact prediction sets, especially in multiclass settings.

### Top-k CP

Top-k CP differs from the other methods as it produces prediction sets of fixed size for every test observation. Instead of using probabilities directly, it uses the rank of the true class as the non-conformity score. For each calibration sample, the model outputs probabilities that are sorted from highest to lowest. The non-conformity score is simply the position of the true class in this ordered list. The threshold used to build prediction sets is obtained by taking the 1- α quantile of these scores. When applied to a test sample, the model again produces a ranked list of classes. The prediction set is then constructed by taking the top-k classes where k corresponds to the calibrated threshold.

### Class-conditional CP

Class-conditional CP modifies the LAC method by computing separate quantiles of non-conformity scores for each stenosis grade class, enabling the generation of class-specific thresholds tailored to different categories. In particular, instead of computing a single 1- α quantile across all calibration samples, the method computes separate quantiles for each class. For every class, the non-conformity scores are summarised independently, producing class-specific thresholds reflecting potential differences in difficulty and class imbalance. At test time, the process is the same as in LAC but a class is included in the prediction set if its score falls below its own class-specific threshold. As a result, prediction sets can vary in size and composition in a way that better adapts to the characteristics of each stenosis grade.

### Evaluation

For each method and significance level, results were aggregated across all bootstrap iterations to compute different statistics (mean and confidence intervals) of both empirical coverage and prediction set sizes. These metrics were computed both on the full test and for each CCS class. In particular, the coverage on the full test set is useful to check if the empirical coverage was consistent with the expected coverage (1 - α) while the CCS classes’ conditional coverage is to investigate if the empirical coverage level was high enough for each stenosis grade. Moreover, since the prevalence of stenosis gradings is different across vertebral levels (Fig. 2 top) a level-specific analysis was implemented to explore for each level (L2/L1 to S1/L5) the empirical coverage and prediction set size of each stenosis grade. To facilitate the reading, this last analysis was only performed for α = 0.15 and used the best-performing method in terms of high coverage and small prediction set size across the stenosis gradings. Regarding the CP evaluation, prediction set size is important to understand the confidence of the model in the prediction: larger sets indicate higher uncertainty while smaller ones reflect more confident predictions. This comprehensive evaluation enables an assessment of the robustness and stability of each CP method across various dataset splits. By analyzing different α levels, the study explores the relationship between coverage, prediction set sizes, and α values, providing insights to help readers select the most suitable α for their specific use case. Regarding the implementation, SpineNet model is freely available on GitHub^13^ and it is written in Python using PyTorch library. The code to apply CP has been developed using Python 3.11. The main libraries used were pandas to load and process the data, numpy to compute CP, and seaborn and matplotlib for plotting. The code to run SpineNet and apply CP to the softmax outputs is available on our GitLab (https://gitlab.ethz.ch/BMDSlab/publications/low-back/conformal-prediction-spinenet) and on the corresponding author’s GitHub page (https://github.com/ancina/conformal-prediction-spinenet-github).

## Supplementary Information

Below is the link to the electronic supplementary material.

Supplementary Material 2

Supplementary Material 2

Supplementary Material 3

Supplementary Material 4

## Data Availability

The datasets generated and/or analysed during the current study are not publicly available due to privacy and ethical restrictions. The code to run the analysis as well as create the figures is available on our GitLab repository [https://gitlab.ethz.ch/BMDSlab/publications/low-back/conformal-prediction-spinenet](https:/gitlab.ethz.ch/BMDSlab/publications/low-back/conformal-prediction-spinenethttps:/gitlab.ethz.ch/BMDSlab/publications/low-back/conformal-prediction-spinenet) and (https:/gitlab.ethz.ch/BMDSlab/publications/low-back/conformal-prediction-spinenethttps:/gitlab.ethz.ch/BMDSlab/publications/low-back/conformal-prediction-spinenet) on the corresponding author’s GitHub repository (https://github.com/ancina/conformal-prediction-spinenet-github).
