# Supplementary figures and images for: Quantifying central canal stenosis prediction uncertainty in SpineNet with conformal prediction
Source: Sci Rep. 2026 Jan 10;16:4963. doi: 10.1038/s41598-026-35343-6 (PMC12876996; doi:10.1038/s41598-026-35343-6)

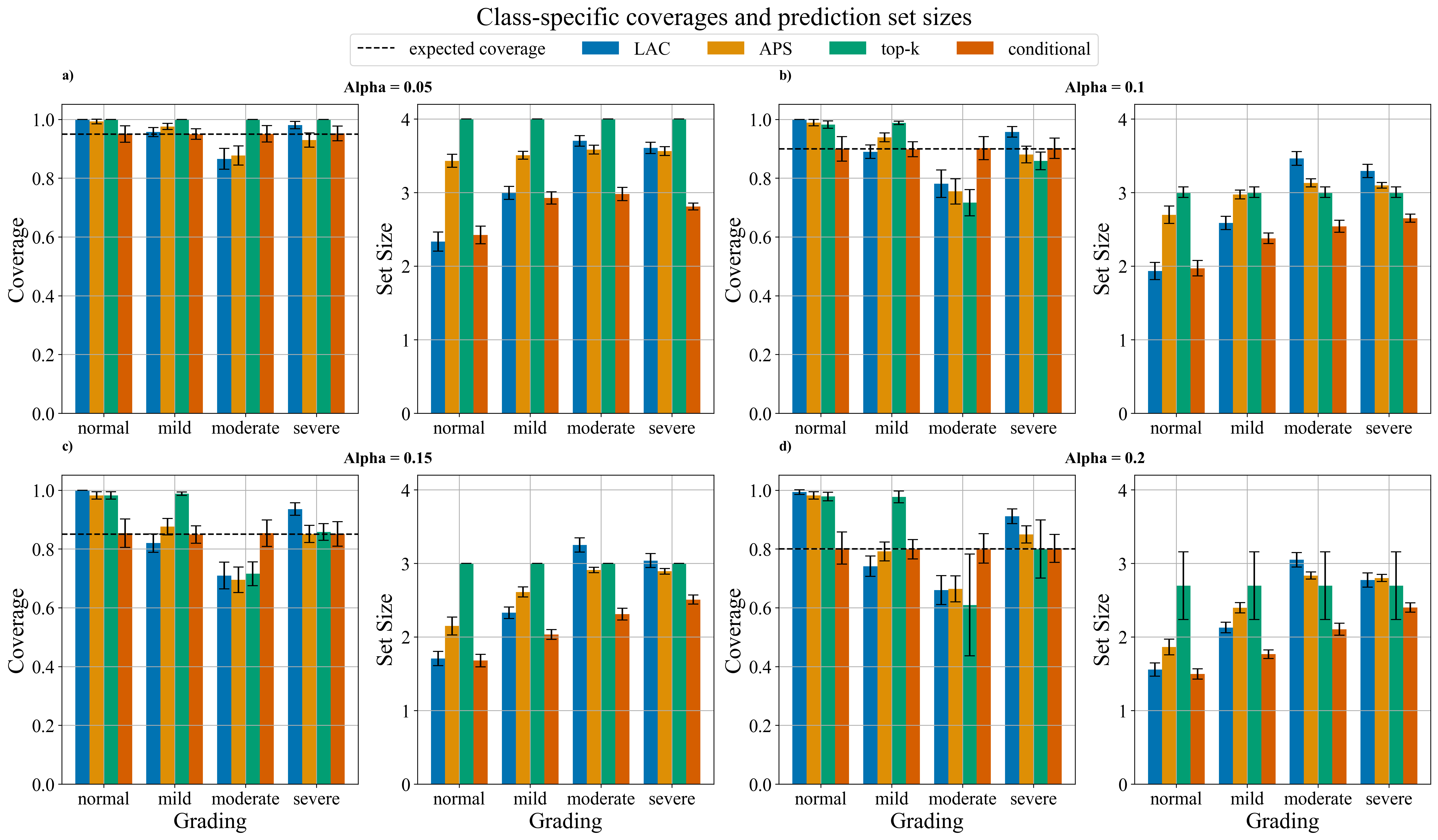

Supplement: Supplementary file 1 — Supplementary Material 2 [file 41598_2026_35343_MOESM1_ESM.png]

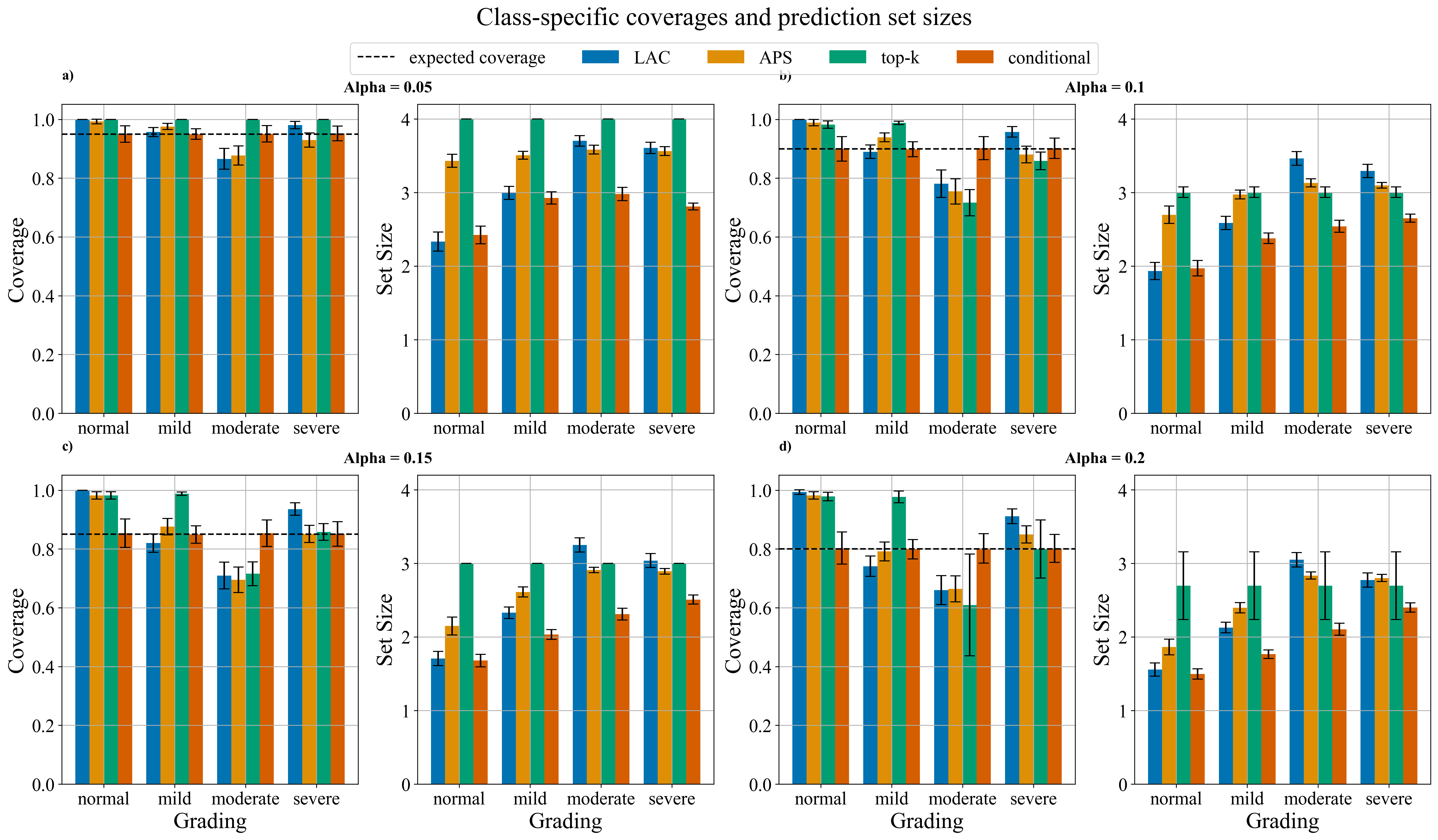

Supplement: Supplementary file 2 — Supplementary Material 2 [file 41598_2026_35343_MOESM2_ESM.png]

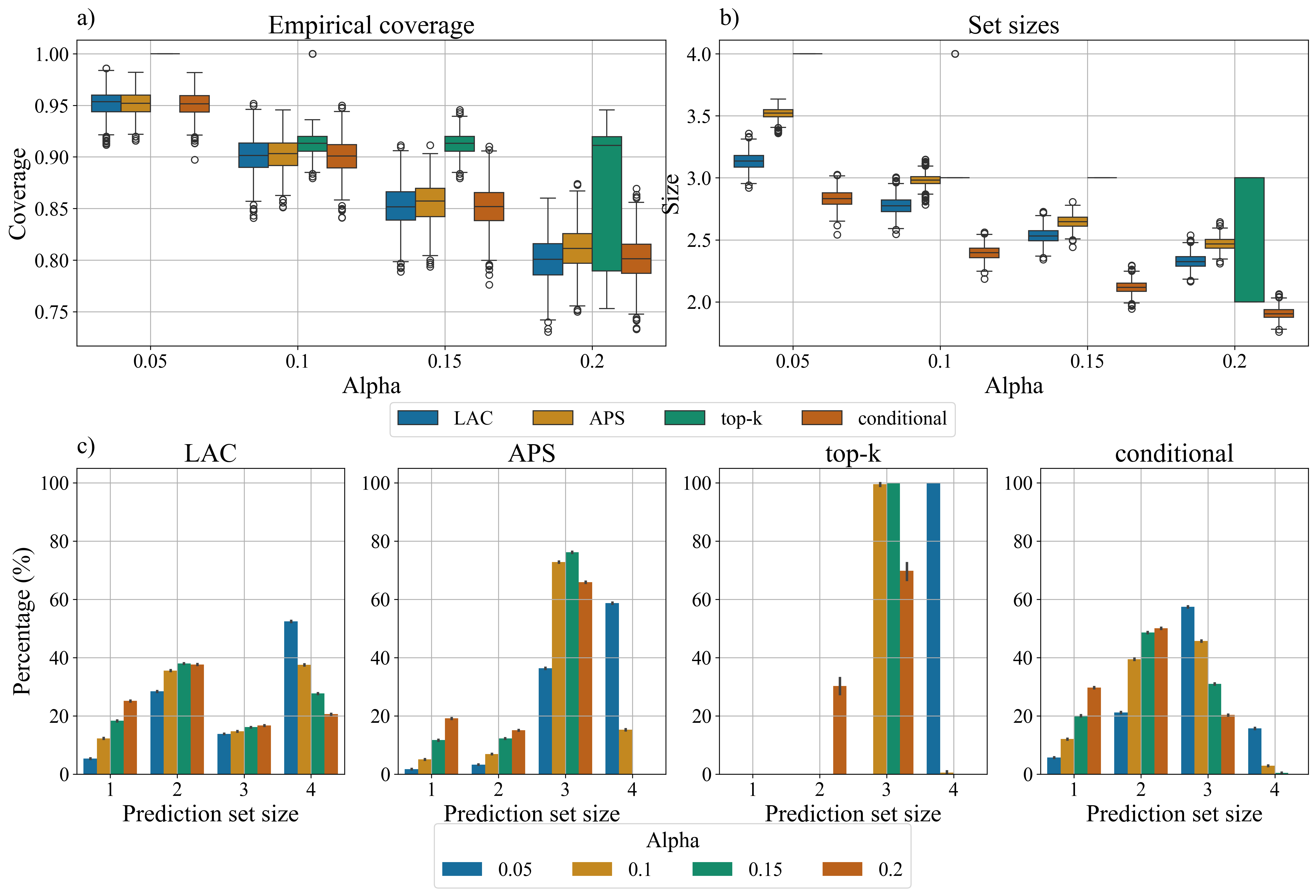

Supplement: Supplementary file 3 — Supplementary Material 3 [file 41598_2026_35343_MOESM3_ESM.png]

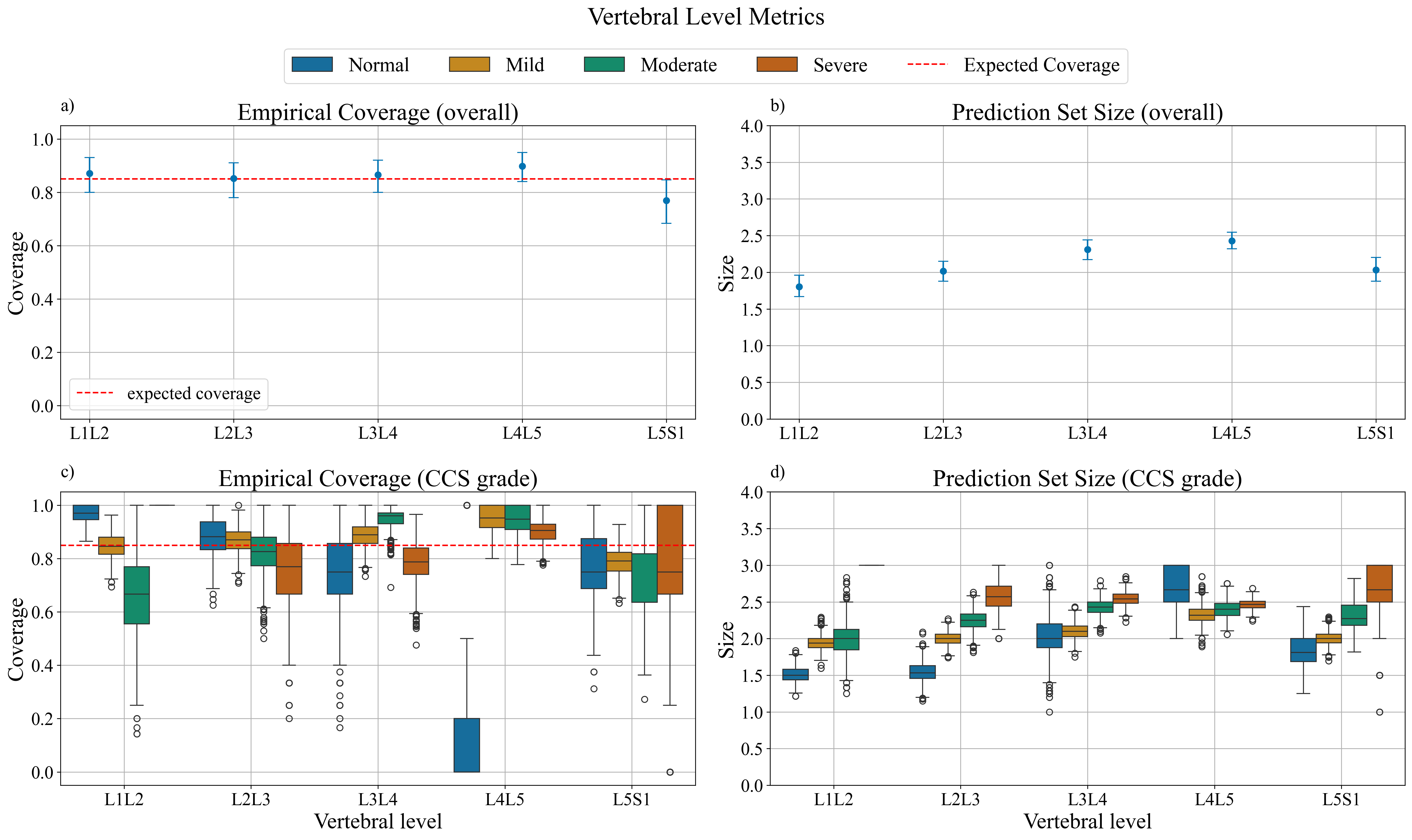

Supplement: Supplementary file 4 — Supplementary Material 4 [file 41598_2026_35343_MOESM4_ESM.png]
